# From Submerged Cultures to 3D Cell Culture Models: Evolution of Nasal Epithelial Cells in Asthma Research and Virus Infection

**DOI:** 10.3390/v13030387

**Published:** 2021-02-28

**Authors:** Malik Aydin, Ella A. Naumova, Aliyah Bellm, Ann-Kathrin Behrendt, Federica Giachero, Nora Bahlmann, Wenli Zhang, Stefan Wirth, Friedrich Paulsen, Wolfgang H. Arnold, Anja Ehrhardt

**Affiliations:** 1 Laboratory of Experimental Pediatric Pneumology and Allergology, Center for Biomedical Education and Research, School of Life Sciences (ZBAF), Faculty of Health/School of Medicine, Witten/Herdecke University, Alfred-Herrhausen-Str. 50, 58448 Witten, Germany; 2 Children’s Hospital, Helios University Hospital Wuppertal, Center for Clinical and Translational Research (CCTR), Witten/Herdecke University, 42283 Wuppertal, Germany; stefan.wirth@helios-gesundheit.de; 3 Department of Biological and Material Sciences in Dentistry, Faculty of Health, Witten/Herdecke University, 58455 Witten, Germany; ella.naumova@uni-wh.de (E.A.N.); wolfgang.arnold@uni-wh.de (W.H.A.); 4 Helios Hospital Krefeld, Children’s Hospital, Teaching Hospital of RTWH University Hospital Aachen, 47805 Krefeld, Germany; aliyah.j.sanders@gmail.com; 5 Pediatric Rheumatology and Immunology, Department of Pediatrics, University Medicine Greifswald, 17475 Greifswald, Germany; ann-kathrin.behrendt@med.uni-greifswald.de; 6 Laboratory of Clinical Molecular Genetics and Epigenetics, Center for Biomedical Education and Research, School of Life Sciences (ZBAF), Faculty of Health, Witten/Herdecke University, 42283 Wuppertal, Germany; federica.giachero@uni-wh.de; 7 Institute of Virology and Microbiology, Center for Biomedical Education and Research (ZBAF), Department of Human Medicine, Faculty of Health, Witten/Herdecke University, 58453 Witten, Germany; nora.bahlmann@uni-wh.de (N.B.); wenli.zhang@uni-wh.de (W.Z.); anja.ehrhardt@uni-wh.de (A.E.); 8 Institute of Functional and Clinical Anatomy, Friedrich Alexander University Erlangen-Nuernberg, 91054 Erlangen, Germany; friedrich.paulsen@fau.de; 9 Department of Topographic Anatomy and Operative Surgery, Sechenov University, 119146 Moscow, Russia

**Keywords:** epithelial cells, nasal epithelial cells, bronchial epithelial cells, submerged, 3D cell culture, spheroids, air–liquid-interface, virus, asthma, culture techniques

## Abstract

Understanding the response to viral infection in the context of respiratory diseases is of significant importance. Recently, there has been more focus on the role of the nasal epithelium in disease modeling. Here, we provide an overview of different submerged, organotypic 3D and spheroid cell culture models of nasal epithelial cells, which were used to study asthma and allergy with a special focus on virus infection. In detail, this review summarizes the importance, benefits, and disadvantages of patient-derived cell culture models of nasal- and bronchial epithelial cells, including a comparison of these cell culture models and a discussion on why investigators should consider using nasal epithelial cells in their research. Exposure experiments, simple virus transduction analyses as well as genetic studies can be performed in these models, which may provide first insights into the complexity of molecular signatures and may open new doors for drug discovery and biomarker research.

## 1. Introduction

Asthma remains of the most important airway diseases worldwide, being associated with a significant number of people suffering from this illness [1,2,3]. The immunology of asthma is composed of distinct molecular pathways involving cytokines, chemokines, and transcription factors that orchestrate the signaling between innate and adaptive immunity [2,4,5]. Distinct allergy/asthma cohorts (including cohorts from birth on) have been previously established and studies in these cohorts performed with the common aim of understanding the clinical and molecular determinants of childhood asthma and allergies [6,7,8,9,10].

Viral infections can contribute to asthma development and allergic sensitization [11,12]. Previous studies on respiratory syncytial virus (RSV) have shown that children with prior RSV bronchiolitis who presented with chronic persistent symptoms had an increased risk for allergic sensitization and asthma [13,14]. Patients with underlying diseases, i.e., children with chronic bronchitis or bronchial asthma, are additionally more susceptible to severe symptoms due to viral infection [15,16]. In particular, RSV, rhinovirus, influenza virus, bocavirus, but also adenoviruses and coronavirus may play a role not only in exacerbation but also in disease modeling in asthmatics [17,18,19,20,21,22]. To understand the pathogenesis of respiratory illness due to viruses, to analyze the actual diagnostic standards, or to develop novel treatment options for patients suffering from asthma, it is essential to study the molecular effects of such viruses in more detail.

Cell culture models are indispensable in precisely answering these questions [23,24]. In particular, primary human nasal epithelial and bronchial epithelial cells enable the study of important molecular characteristics of viral infection and have been extensively used to reveal defining features of numerous viruses, including the recently emergent coronavirus, SARS-CoV-2 [25,26,27,28]. For the implementation of such experiments, observations from cell cultures (e.g., submerged, organotypic, spheroids, chip-based methods, organoids, etc.) derived from normal and diseased patients may be important to study the molecular role of viruses and to define potential “viral biomarkers” for clinical routine. Considering the importance of and the lack of data regarding nasal epithelial cells, with this review, we aim to discuss the necessity of cell culture models of nasal epithelial cells in respiratory research with a special focus on viral infection in asthma, presenting our experiences and summarizing the advantages and pitfalls when working with this cell group.

## 2. A General Overview on Cell Culture Techniques in Respiratory Medicine

Since the beginnings of scientific research, significant effort has been dedicated towards keeping cells alive ex vivo [29]. The first immortal human cell line, originating from the cervical carcinoma of Henrietta Lacks (HeLa cells, albeit without the direct consent of Miss Lacks) was an enormous breakthrough and success for experimental medicine [30,31]. Increasingly, many immortal cell lines as well as primary mammalian cells find an important place in the laboratory. Submerged cell cultures of primary bronchial epithelial cells are often used to perform in vitro experiments related to the respiratory system, e.g., testing the effects of monoclonal antibodies, but also of air pollution, diesel, and allergens [22,32,33]. The main disadvantage of this submerged culture model is that the cells lose their ciliated phenotype and do not replicate human respiratory physiology, in fact [34,35,36,37]. Since the early 1970s, studies utilizing bronchial epithelial cell cultivation techniques have been of great significance for researchers [38,39,40]. Moreover, researchers have now developed new culturing methods that more closely mimics the human physiology [41]. The air–liquid interface (ALI) culture model is one of the first important cell culture technique in respiratory research, where mucociliary differentiation and mucus production in vitro can be achieved [41,42,43,44]. In brief, cells are grown on semipermeable, transwell filters (=cell culture inserts) which are solely exposed to the culture medium from the basolateral surface and the apical surface of the cells are exposed to the air, which enables cells to develop a “pseudostratified morphology” [22,45,46,47]. This work builds on the historically significant groundwork established by Whitcutt and colleagues, who developed the “Whitcutt chamber” for the cultivation of respiratory epithelial cells in 1987. Here, a chamber with a special membrane and a c-like ring were used to similar effect as the current ALI model [48].

Efforts are currently aimed at achieving maximal physiological conditions that accurately recapitulate molecular responses in vivo. Certainly, so-called 3D cultures represent an important milestone in experimental research [49,50]. Animal experiments can provide important molecular answers to some clinical translational questions [51,52]. However, due to the fact that human airway physiology and lung diseases such as asthma cannot be perfectly imitated in animal models, e.g., in mice [53], 3D models represent an important methodology in respiratory research to pursue potential translational hypotheses and drug response studies [54,55]. Moreover, classical cell cultures are typically established from one cell line or cell population although the infectious process involves many cell subsets in vivo [56]. Here, tissue engineering/3D models may overcome this limitation [56]. Hence, the common goal of 3D-cultures is that cell aggregates embedded in a tight space, e.g., in matrices or “scaffold” materials (e.g., the growth on agar, gelatin, collagen, etc.), develop so-called collective integrity, in which cells communicate closely with each other beyond external (constant change of cell culture media) or internal (material of cell culture flasks) confounding factors [37,49,57,58,59]. Furthermore, spheroid cultures represent an additional method for the rapid application of 3D-cultures and may also be prepared from nasal epithelial cells [60]. While bronchial- and nasal epithelial cells may dedifferentiate when seeded as a monolayer in flasks, 3D-spheroids seeded in suspension medium do not actually suffer from this limitation [61,62,63,64,65]. In addition to ALI [41,45,66,67], bronchial- and nasal epithelial spheroid cultures [62,68,69], further 3D-models [54], precision cut lung slices [70,71], bronchial rings [72,73], and scaffold-based models [49,74,75,76] as well as organoids [77,78] etc. have been already established from lung and nasal epithelium to this end. Interestingly, organ-like tissue structures can be also developed from pluripotent stem cells, which are currently referred to as mini-organs or organoids. These are self-organizing 3D-models for extended period culturing, enabling the study of disease modeling in dishes through their similar in vivo morphology and physiology [79,80,81,82].

In summary, in review of the literature, bronchial epithelial but also increasingly nasal epithelial cell culture models are essential for respiratory research [78,83,84,85]. The work with spheroids and organoids, particularly after infection, could provide a closer look into the molecular response, which has not yet to be fully explored.

## 3. Bronchial Epithelial versus Nasal Epithelial Cell Culture: Benefits and Drawbacks

Although culturing primary mammalian cells derived from patients is established in the platforms mentioned above, the collection of bronchial epithelial cells is more difficult to implement in practice compared to nasal epithelial cells [86,87]. Generally, healthy children and adolescents do not routinely undergo invasive bronchoscopic interventions. Particularly in young children, these examinations are reserved, for example, for foreign body aspiration, or to clarify chronic cough due to lung pathology, hemoptysis, or suspected neoplasia [88,89,90]. Indeed, based on our experiences, patients requiring bronchoscopic examinations are mostly elderly patients with a longstanding medical history. Thus, compared to healthy cells, these cells may have a different morphology that can significantly influence the experimental outcomes. For example, a 60-year-old patient with a chronic nicotine abuse or a chronic lung disease will certainly show a different cell morphology than that of a healthy 2-year-old child with a no complex medical history.

Although a few authors have previously described the possibility of performing bronchial brushing in patients [91,92], there must nevertheless be an important indication to perform invasive examinations including bronchoscopy. Additionally these tests are associated with great medical effort as well as significant risk of complications during the procedure [93]. Moreover, the sampling from patients with, e.g., severe asthma, is extremely difficult to implement through bronchoscopy [93]. It is generally known that it is difficult to recruit a homogenous patient group to exclude inter- and intraindividual differences between subjects. To exclude potential bias, an appropriate study design should be prepared accordingly [54]. Commercially purchased cells from deceased individuals are an alternative possibility; but death itself may be associated with different molecular mechanisms resulting again in decreased similarity to the in vivo reality. In addition, in the immortal cell lines that can be commercially purchased [87], the question arises whether these cells present an in vivo like response similar to that of pediatric patients with viral infection and exacerbated asthma.

**Figure 1:**
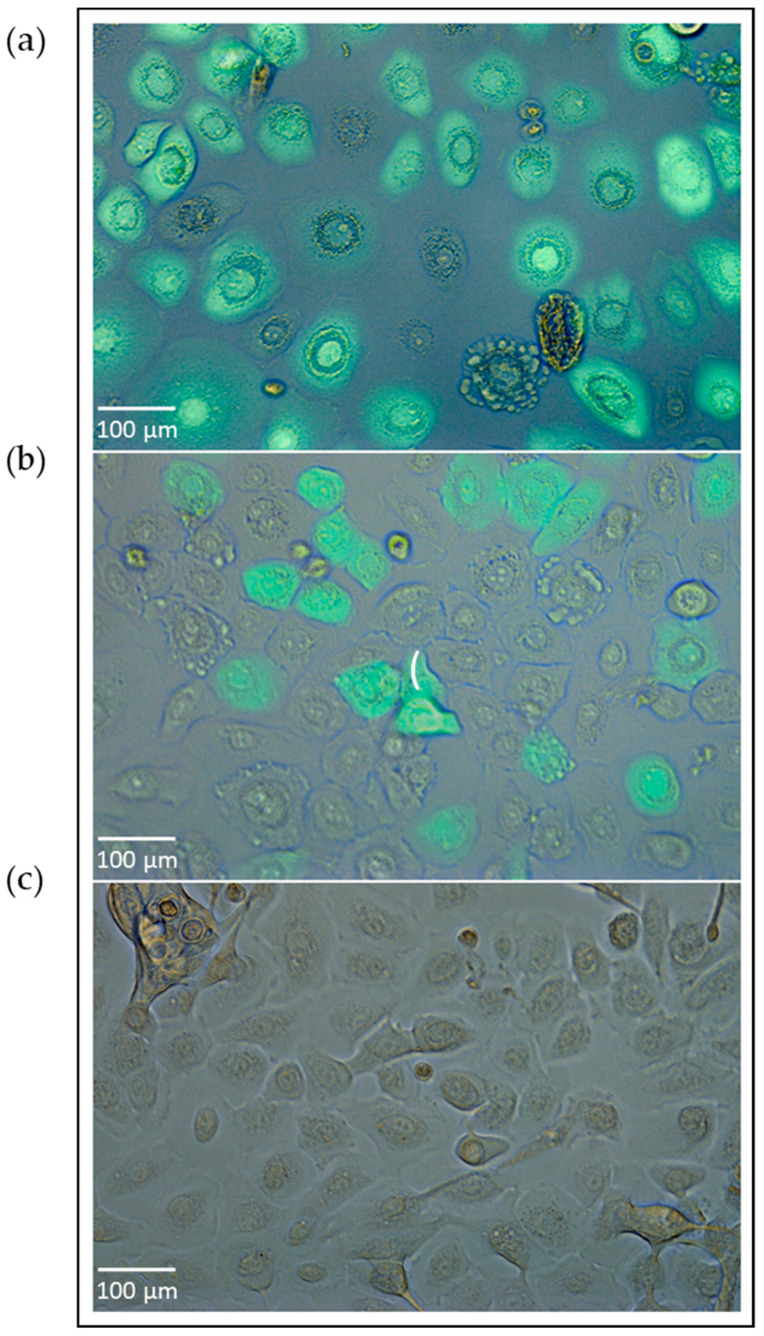
The submerged culture of nasal epithelial cells transduced with adenovirus. The cultivation of nasal epithelial cells derived from a healthy subject from our pediatric exacerbation study cohort [96]. Nasal epithelial cells were seeded in an amount of 20,000 cells per well and resuspended in BEGM^TM^ medium (Lonza, Basel, Switzerland) in collagen-coated 24-well culture plates prior to virus transduction. The next day, previously established recombinant adenovirus type 5 encoding green fluorescent protein [97] was added in different virus particle concentrations (vpc) (e.g., 1 × 10^8^ vpc), incubated for one day upon viral transduction, and the transduction efficiency was analyzed through immunofluorescence microscopy. The subfigures (**a**,**b**) represent virus transduction efficiencies in nasal epithelial cells directly correlating with virus transduction rates. The picture (**c**) shows the nasal epithelial cells without viral transduction (untreated, wildtype cells). This experiment was separately performed for this manuscript, correlates thematically with [22], and was not previously published elsewhere.

As a potential alternative to bronchial epithelial cells, nasal epithelial cells can be obtained from children and adolescents with exacerbated asthma or chronic bronchitis as well as from healthy children and adults through nasal brushing procedure without significant medical interventional effort or invasiveness [22,86,94]. Nasal brushing also offers the additional benefit of longitudinal collection of epithelial cells over different time points [93]. Furthermore, nasal epithelial cells do not mostly need to undergo any enzymatic digestion prior culturing [93]. Thus, cells can be directly seeded for experimental analyses, e.g., in submerged cultures/monolayers in flasks [93]. The downside to this, however, is that the cell pellets may also include other cell types (e.g., fibroblasts, erythrocytes, etc.). Thus, a flow cytometric characterization of the cells, for example, should be performed to determine the purity of the cultures. Based on previous flow cytometry protocols characterizing bronchial epithelial cells, antibodies targeting CD45, CD326 and Pan-Cytokeratin can be used to determine the purity of the nasal epithelial cells [22,95]. If the culture consists of multiple cell types, the nasal epithelial cells can be also sorted with a fluorescence-activated cell sorter, and a few days thereafter, basic exposure-to-effect analyses or co-culture experiments can be easily performed. For example, to study the permissiveness of nasal epithelial cells for virus transduction, Figure 1 exemplifies a submerged culture of primary nasal epithelial cells infected with a reporter gene-expressing adenovirus.

**Figure 2:**
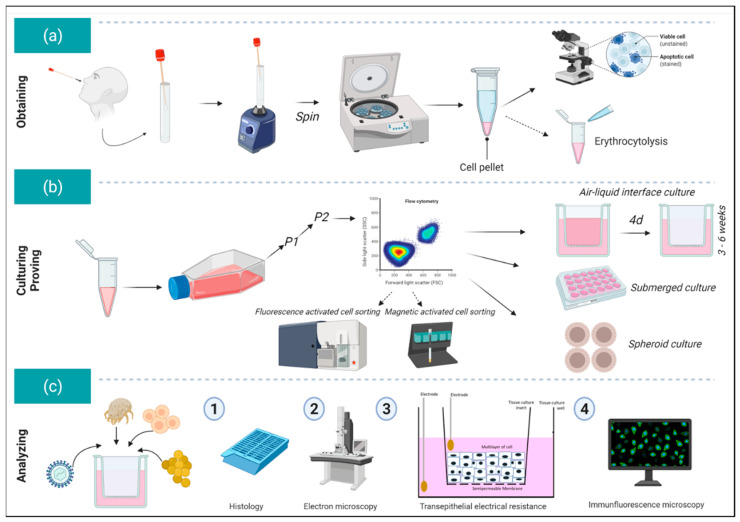
An overview of the workflow for analyzing primary human nasal epithelial cells. (**a**) After careful collection of the nasal epithelial cells from the nasal cavity using a cytology or interdental brush, cells are transferred to a 15 mL tube and shaken several times. The brush is then removed, and the cells are centrifuged. The cells are checked for viability using a Neubauer counting chamber using trypan blue or Türk’s solution (purchased from Sigma Aldrich). If necessary, erythrocytolysis can be also performed separately. (**b**) The cells are then seeded into collagen-coated T25/T75 flasks in, for example, BEGM^TM^ medium. After two passages, flow cytometric characterization of the cells can be performed if needed. If the cultures lack purity, separation by Magnetic-activated cell sorting (MACS) or Fluorescence-activated cell sorting (FACS) can be also performed (usually these steps are not necessary). Afterwards the nasal epithelial cells are either transferred into air–liquid interface cultures (ALI) (approximately 9–11 × 10^4^ cells per 1.12 cm^2^ or 2–3 × 10^4^ cells per 0.33 cm^2^ cell culture inserts = transwells), or directly seeded in submerged cultures. For certain questions, nasal epithelial cells can be also seeded for spheroid cultures (circa 1000 cells per U-bottom well). (**c**) Different provocation experiments or co-cultures can be performed with the cultures (here schematic presentation for ALI cultures) and subsequently, the morphology of the cells can be studied through histology and electron microscopy [22]. By means of transepithelial electrical resistance measurement, the integrity of the cell–cell interaction can be measured, or the permissiveness can be investigated through immunofluorescence microscopic analysis. This content of this figure correlates thematically with [22] and was not previously published elsewhere. This figure was created with www.biorender.com (accessed on 27 February 2021).

**Figure 3:**
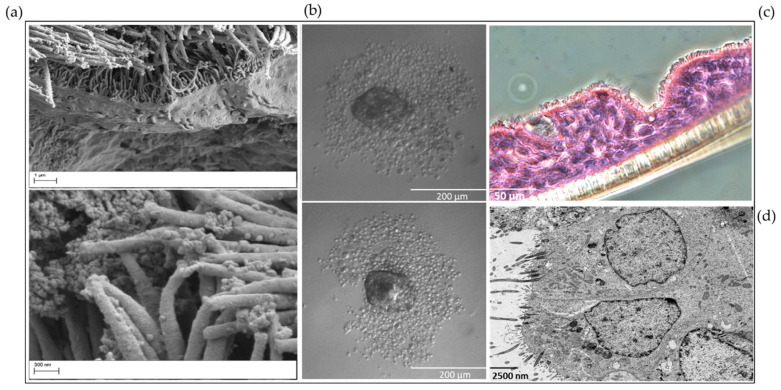
The morphology of nasal epithelial cells. (**a**) Scanning electron microscopic imaging of organotypic 3D nasal epithelial cell cultures derived from young children with asthma or healthy subjects (left top: ×4.00 K; left bottom: ×9.45K, in different sizes). These images show the morphology of the nasal epithelial cell culture with cilia, the integrity of the cell–cell-interaction, and the glycocalyx structure. The samples were prepared for imaging with Zeiss Sigma VP SEM (Zeiss, Oberkochen, Germany) scanning electron microscope at 2 kV acceleration voltage using the in-lens and SE detectors. (**b**) Nasal epithelial cells were seeded for the submerged spheroid culture technique. Cells derived from a healthy subject were seeded in a 96-well plate at circa 1000 cells per well in U-Bottom plate (×10 magnification, size 200 µm) receiving PneumaCult^TM^ ALI medium (STEMCELL^TM^ Technologies, Vancouver, Canada). After reaching a determined cell size, the spheroid culture can be used for distinct experimental questions. This experiment was separately performed and correlates thematically with [22]. (**c**) ALI culture derived from a healthy subject. After passaging nasal epithelial cells from the 2D submerged into the organotypic 3D ALI culture model, samples were prepared for histological analyses. This figure presents the cilia morphology of the cultures and the cell–cell integrity in PAS reaction. The ciliary epithelium is precisely imaged (×10 magnification, size 50 µm). (**d**) Transmission electron microscopic (TEM) analysis of nasal epithelial ALI cultures (×20,000 magnification, size 2.500 µm). The aim of this additional method and figure is to present the cell–cell–contacts (tight junctions), cilia and organelles through TEM to study the effectiveness of the cell culture model. These methods/pictures were separately performed, correlate thematically with [22] and were not previously published elsewhere.

Air–liquid interface and spheroid cell cultures can be also prepared from nasal epithelial cells and provide the possibility of analyzing the effects of external stimuli on cell–cell integrity and/or on cell–matrix interaction [80,98]. An overview of the workflow for obtaining, culturing, and analyzing nasal epithelial cells is provided in Figure 2, while Figure 3 shows different types of cultures derived from nasal epithelial cells. 

The derivation of these differentiated models with nasal epithelial cultures share many of the same downsides as bronchial epithelial cultures including similar culture conditions [66,94,99,100,101], for example, growth arrest [45,101,102], sensitivity to trypsin [41,100,101,103,104], risk of contamination [41,66,100,101,104,105] and a multi-week differentiation process [42,66,101], as well as high cost. Recently, Broadbent and colleagues tested different cell culture media using Promocell and PneumaCult media and analyzed the effects of RSV in primary nasal epithelial cells [106]. They showed that there were no significant differences in the viral response in vitro using both cell culture media. When using PneumaCult, the number of the pseudostratified cells was higher than using Promocell [106]. Furthermore, Luengen et al. (2020) [107] compared different cell culture media to study the mucociliary differentiation of respiratory epithelial cells in their entire cohort. They included cells from subjects in the age range of 10 to 40 years (excluding one subjects being between 50 and 60 years of age) and tested four different cell culture media including (1) mAir, which is a modified version of AECBM (purchased from PromoCell), DMEM and different supplements, (2) PneumaCult-ALI from STEMCELL^TM^ Technologies, furthermore, (3) Epi was prepared using MucilAir culture medium (purchased from Epithelix) with tranexamic acid and (4) EMM was prepared using MucilAir Culture medium, EGM2 an tranexamic acid. For the detailed media prescription, we kindly refer to [107]. They interestingly concluded that the mAir media from PromoCell provided better ciliary differentiation than other three cell culture media. The authors also discuss a significant subject-variability in their study protocol, and a potential age- and clinical history-dependency [107]. Generally speaking, nasal and bronchial epithelial cells present very similar morphology and histology, among other similarities [68,94].

As early as 1990, Devalia et al. [94] compared bronchial- and nasal epithelial cells. They were able to show that nasal epithelial cells have the same morphology and similar in vitro cell culture growth and ciliary activity as bronchial epithelial cells, and that both cell populations are suitable for airway tract studies [94]. In their study, Roberts and colleagues [108] analyzed the molecular effects of human rhinovirus as well in-vitro-IL-13 treatment in nasal and bronchial epithelial cells in submerged and ALI cultures. Based on their results, the two cell populations presented an almost identical response, so that the authors concluded that nasal epithelial cells may be a potential laboratory surrogate for bronchial epithelial cells. Thus, due to a few significant benefits of nasal epithelial cells over bronchial epithelial cells, nasal epithelial cells will increasingly play a large role in research laboratories.

## 4. The Application of Nasal Epithelial Cell Culture Models in Asthma and Virus Research

The nasal cavity has long been classified as a physical barrier organ and was assumed to be merely responsible for filtering and cleaning processes [109,110]. When the integrity of this barrier is impaired, the risk for chronic rhinosinusitis is increased [111]. For some years now, however, researchers have been increasingly focused on this organ and analyzed its role in the pathogenesis of allergic rhinoconjunctivitis, asthma and virus infection [28,112,113,114]. Several, previously published studies showed that nasal epithelial cells of patients with chronic rhinitis have connections to disease development on distinct intracellular levels (e.g., posttranscriptional, and posttranslational modifications). In order to work in systems most closely resembling in vivo conditions, patient-derived samples are an invaluable material for answering diverse research questions [115,116].

Jackson et al. [117] investigated the impact of rhinoviruses on type 2 inflammation and found that in vivo provocation with rhinovirus-16 can lead to an increase of IL-33 and type 2 cytokine concentrations, particularly in asthmatics. In addition, the authors observed that an in vitro stimulation of bronchial epithelial cells by rhinovirus-16 resulted in a significant increase of IL-33 [117]. The work of Pech and colleagues [118] analyzed the role of rhinovirus infection in a submerged culture model of nasal epithelial cells (derived from a multicenter pediatric asthma cohort) and observed important methylation patterns in their study. Furthermore, Vanders et al. [114] demonstrated the role of rhinovirus-43 and influenza virus (H3N2) in nasal epithelial cells of pregnant asthmatics. This study revealed diverse increases in cytokine and chemokine profiles and explained that nasal epithelial cells can also serve as an important method for the analyses of immune responses in pregnant asthmatics in vitro [114]. Yamaya and colleagues [119] studied the in vitro effects of clarithromycin and rhinovirus-13 infection on the nasal epithelial cells of asthmatics. Among other findings, they observed that prior in vitro antibiotic therapy is associated with a reduction of viral titers and cytokine concentrations. While this study struggled with certain limitations, these pilot results provide important first connections between infection and cell culture models in asthma research [119]. Table 1 summarizes the research article which include the search items nasal epithelial cell cultures, asthma, and virus.

Taken together, bronchial epithelial but also increasingly nasal epithelial cell culture models are of increasing importance for respiratory research. Based on the literature, submerged and ALI cultures are already firmly established, and differentially evaluated models are in constant use. Work regarding spheroids and organoids in asthma and infection models can provide a closer look at in vitro responses to viral infection. The work with ALI and spheroid cultures, as well as precision cut lung slices, and organoid models in respiratory research [70,71,78,83,84] may enable to perform virus transduction experiments which would provide a closer look into the still largely unexplored molecular virus responses ex vivo.

## 5. Outlook

The cell culture methodology of bronchial epithelial and nasal epithelial cells has undergone a rapid development. Experiments that utilize these cells add an important contribution to research platform. Efforts are therefore being made to perform nearly in vivo-like experiments under similar physiological conditions. Nasal epithelial cells currently represent a potential alternative to bronchial epithelial cells in respiratory research. Despite the establishment of numerous cell culture technologies, it is of great importance to apply the results derived from these complex cell culture models and to translate them into the clinics. It will be exciting to observe whether nasal organoids will be effective research tools and in which ways they will molecularly react to allergens, noxious agents such as diesel gases or microbial endotoxins.

## Figures and Tables

**Table 1:** Overview of published research articles associated with nasal epithelial cell culture models, asthma, and virus infections.

Research Article	Virus	Disease	Type of Nasal Epithelial Cell Culture
Pech and colleagues (2018) [118] Rhinovirus infections change DNA methylation and mRNA expression in children with asthma	RV ^1^ 16	Asthma	submerged
Yamaya and colleagues (2020) [119] Clarithromycin decreases rhinovirus replication and cytokine production in nasal epithelial cells from subjects with bronchial asthma: effects on IL-6, IL-8 and IL-33	RV 14	Asthma	submerged
Lopez-Souza and colleagues (2009) [120] In vitro susceptibility to rhinovirus infection is greater for bronchial than for nasal airway epithelial cells in human subjects	RV 16	Asthma	ALI ^2^
Spann and colleagues (2014) [121] Viral and host factors determine innate immune responses in airway epithelial cells from children with wheeze and atopy	RSV ^3^ & HMPV ^4^	Wheeze/Atopy	submerged
McErlean and colleagues (2014) [122] Human rhinovirus infection causes different DNA methylation changes in nasal epithelial cells from healthy and asthmatic subjects	RV	Asthma	submerged
Baturcam and colleagues (2017) [112] Human Metapneumovirus Impairs Apoptosis of Nasal Epithelial Cells in Asthma via HSP70	RSV & HMPV	Asthma	submerged
Vanders and colleagues (2019) [114] Nasal epithelial cells to assess in vitro immune responses to respiratory virus infection in pregnant women with asthma	RV 43 & H3N2 ^5^	Asthma	submerged and ALI
Aydin and colleagues (2020) [22] House Dust Mite Exposure Causes Increased Susceptibility of Nasal Epithelial Cells to Adenovirus Infection.	AdV ^6^	Asthma	submerged and ALI
Yamaya and colleagues (2020) [123] Inhibitory effects of glycopyrronium, formoterol, and budesonide on coronavirus HCoV-229E replication and cytokine production by primary cultures of human nasal and tracheal epithelial cells	HCoV-229E ^7^	among others Asthma	ALI

^1^ RV = (human)rhinovirus; ^2^ ALI = air–liquid interface; ^3^ RSV = Respiratory syncytial virus; ^4^ HMPV = (human)Metapneumovirus; ^5^ H3N2 = Influenza A virus subtype H3N2; ^6^ AdV = Adeno-virus; ^7^ HCoV-229E = Coronavirus 229E.

## Data Availability

The data presented in this study are available on request from the corresponding author.

## References

[1] Braman S.S. (2006). The global burden of asthma. Chest.

[2] Lambrecht B.N., Hammad H. (2015). The immunology of asthma. Nat. Immunol.

[3] Asher I., Pearce N. (2014). Global burden of asthma among children. Int. J. Tuberc Lung Dis..

[4] Lambrecht B.N., Hammad H. (2012). The airway epithelium in asthma. Nat. Med..

[5] Wenzel S.E. (2012). Asthma phenotypes: The evolution from clinical to molecular approaches. Nat. Med..

[6] Clarisse B., Nikasinovic L., Poinsard R., Just J., Momas I. (2007). The Paris prospective birth cohort study: Which design and who participates?. Eur. J. Epidemiol..

[7] Fuchs O., Genuneit J., Latzin P., Buchele G., Horak E., Loss G., Sozanska B., Weber J., Boznanski A., Heederik D. (2012). Farming environments and childhood atopy, wheeze, lung function, and exhaled nitric oxide. J. Allergy Clin. Immunol..

[8] Fuertes E., Standl M., Cyrys J., Berdel D., von Berg A., Bauer C.P., Kramer U., Sugiri D., Lehmann I., Koletzko S. (2013). A longitudinal analysis of associations between traffic-related air pollution with asthma, allergies and sensitization in the GINIplus and LISAplus birth cohorts. Peer J..

[9] Illi S., von Mutius E., Lau S., Niggemann B., Gruber C., Wahn U., Multicentre Allergy Study g. (2006). Perennial allergen sensitisation early in life and chronic asthma in children: A birth cohort study. Lancet.

[10] Lau S., Nickel R., Niggemann B., Gruber C., Sommerfeld C., Illi S., Kulig M., Forster J., Wahn U., Groeger M. (2002). The development of childhood asthma: Lessons from the German Multicentre Allergy Study (MAS). Paediatr. Respir. Rev..

[11] Kusel M.M., de Klerk N.H., Kebadze T., Vohma V., Holt P.G., Johnston S.L., Sly P.D. (2007). Early-life respiratory viral infections, atopic sensitization, and risk of subsequent development of persistent asthma. J. Allergy Clin. Immunol..

[12] Loss G.J., Depner M., Hose A.J., Genuneit J., Karvonen A.M., Hyvarinen A., Roduit C., Kabesch M., Lauener R., Pfefferle P.I. (2016). The Early Development of Wheeze. Environmental Determinants and Genetic Susceptibility at 17q21. Am. J. Respir. Crit. Care Med..

[13] Henderson J., Hilliard T.N., Sherriff A., Stalker D., Al Shammari N., Thomas H.M. (2005). Hospitalization for RSV bronchiolitis before 12 months of age and subsequent asthma, atopy and wheeze: A longitudinal birth cohort study. Pediatr. Allergy Immunol..

[14] Sigurs N., Bjarnason R., Sigurbergsson F., Kjellman B. (2000). Respiratory syncytial virus bronchiolitis in infancy is an important risk factor for asthma and allergy at age 7. Am. J. Respir. Crit. Care Med..

[15] Message S.D., Laza-Stanca V., Mallia P., Parker H.L., Zhu J., Kebadze T., Contoli M., Sanderson G., Kon O.M., Papi A. (2008). Rhinovirus-induced lower respiratory illness is increased in asthma and related to virus load and Th1/2 cytokine and IL-10 production. Proc. Natl. Acad. Sci. USA.

[16] Olenec J.P., Kim W.K., Lee W.M., Vang F., Pappas T.E., Salazar L.E., Evans M.D., Bork J., Roberg K., Lemanske R.F. (2010). Weekly monitoring of children with asthma for infections and illness during common cold seasons. J. Allergy Clin. Immunol..

[17] Christensen A., Kesti O., Elenius V., Eskola A.L., Dollner H., Altunbulakli C., Akdis C.A., Soderlund-Venermo M., Jartti T. (2019). Human bocaviruses and paediatric infections. Lancet Child. Adolesc. Health.

[18] Coverstone A.M., Wang L., Sumino K. (2019). Beyond Respiratory Syncytial Virus and Rhinovirus in the Pathogenesis and Exacerbation of Asthma: The Role of Metapneumovirus, Bocavirus and Influenza Virus. Immunol. Allergy Clin. N. Am..

[19] Docherty A.B., Harrison E.M., Green C.A., Hardwick H.E., Pius R., Norman L., Holden K.A., Read J.M., Dondelinger F., Carson G. (2020). Features of 20 133 UK patients in hospital with covid-19 using the ISARIC WHO Clinical Characterisation Protocol: Prospective observational cohort study. BMJ.

[20] Papadopoulos N.G., Christodoulou I., Rohde G., Agache I., Almqvist C., Bruno A., Bonini S., Bont L., Bossios A., Bousquet J. (2011). Viruses and bacteria in acute asthma exacerbations--a GA(2) LEN-DARE systematic review. Allergy.

[21] Soto-Quiros M., Avila L., Platts-Mills T.A., Hunt J.F., Erdman D.D., Carper H., Murphy D.D., Odio S., James H.R., Patrie J.T. (2012). High titers of IgE antibody to dust mite allergen and risk for wheezing among asthmatic children infected with rhinovirus. J. Allergy Clin. Immunol..

[22] Aydin M., Naumova E.A., Paulsen F., Zhang W., Gopon F., Theis C., Lutz S., Ehrke-Schulz E., Arnold W.H., Wirth S. (2020). House Dust Mite Exposure Causes Increased Susceptibility of Nasal Epithelial Cells to Adenovirus Infection. Viruses.

[23] Bochkov Y.A., Palmenberg A.C., Lee W.M., Rathe J.A., Amineva S.P., Sun X., Pasic T.R., Jarjour N.N., Liggett S.B., Gern J.E. (2011). Molecular modeling, organ culture and reverse genetics for a newly identified human rhinovirus C. Nat. Med..

[24] Zeng H., Goldsmith C.S., Kumar A., Belser J.A., Sun X., Pappas C., Brock N., Bai Y., Levine M., Tumpey T.M. (2019). Tropism and Infectivity of a Seasonal A(H1N1) and a Highly Pathogenic Avian A(H5N1) Influenza Virus in Primary Differentiated Ferret Nasal Epithelial Cell Cultures. J. Virol..

[25] Bergougnoux A., Claustres M., De Sario A. (2015). Nasal epithelial cells: A tool to study DNA methylation in airway diseases. Epigenomics.

[26] Kaneko Y., Kohno T., Kakuki T., Takano K.I., Ogasawara N., Miyata R., Kikuchi S., Konno T., Ohkuni T., Yajima R. (2017). The role of transcriptional factor p63 in regulation of epithelial barrier and ciliogenesis of human nasal epithelial cells. Sci. Rep..

[27] McDougall C.M., Blaylock M.G., Douglas J.G., Brooker R.J., Helms P.J., Walsh G.M. (2008). Nasal epithelial cells as surrogates for bronchial epithelial cells in airway inflammation studies. Am. J. Respir. Cell Mol. Biol..

[28] Sungnak W., Huang N., Becavin C., Berg M., Queen R., Litvinukova M., Talavera-Lopez C., Maatz H., Reichart D., Sampaziotis F. (2020). SARS-CoV-2 entry factors are highly expressed in nasal epithelial cells together with innate immune genes. Nat. Med..

[29] Yao T., Asayama Y. (2017). Animal-cell culture media: History, characteristics, and current issues. Reprod. Med. Biol..

[30] Jones H.W. (1997). Record of the first physician to see Henrietta Lacks at the Johns Hopkins Hospital: History of the beginning of the HeLa cell line. Am. J. Obstet. Gynecol..

[31] Lucey B.P., Nelson-Rees W.A., Hutchins G.M. (2009). Henrietta Lacks, HeLa cells, and cell culture contamination. Arch. Pathol. Lab. Med..

[32] Freishtat R.J., Nino G., Tsegaye Y., Alcala S.E., Benton A.S., Watson A.M., Reeves E.K., Haider S.K., Damsker J.M. (2015). Pharmacologically-induced mitotic synchrony in airway epithelial cells as a mechanism of action of anti-inflammatory drugs. Respir. Res..

[33] Huang Y.C., Leyko B., Frieri M. (2005). Effects of omalizumab and budesonide on markers of inflammation in human bronchial epithelial cells. Ann. Allergy Asthma Immunol..

[34] Cao X., Coyle J.P., Xiong R., Wang Y., Heflich R.H., Ren B., Gwinn W.M., Hayden P., Rojanasakul L. (2020). Invited review: Human air-liquid-interface organotypic airway tissue models derived from primary tracheobronchial epithelial cells-overview and perspectives. Vitro Cell. Dev. Biol. Anim..

[35] Upadhyay S., Palmberg L. (2018). Air-Liquid Interface: Relevant In Vitro Models for Investigating Air Pollutant-Induced Pulmonary Toxicity. Toxicol. Sci..

[36] Jorissen M., Van der Schueren B., Van den Berghe H., Cassiman J.J. (1989). The preservation and regeneration of cilia on human nasal epithelial cells cultured in vitro. Arch. Otorhinolaryngol..

[37] Fennema E., Rivron N., Rouwkema J., van Blitterswijk C., de Boer J. (2013). Spheroid culture as a tool for creating 3D complex tissues. Trends Biotechnol..

[38] Barrett L.A., McDowell E.M., Frank A.L., Harris C.C., Trump B.F. (1976). Long-term organ culture of human bronchial epithelium. Cancer Res..

[39] Lechner J.F., Haugen A., McClendon I.A., Pettis E.W. (1982). Clonal growth of normal adult human bronchial epithelial cells in a serum-free medium. Vitro.

[40] Stoner G.D., Katoh Y., Foidart J.M., Myers G.A., Harris C.C. (1980). Identification and culture of human bronchial epithelial cells. Methods Cell Biol..

[41] Fulcher M.L., Randell S.H. (2013). Human nasal and tracheo-bronchial respiratory epithelial cell culture. Methods Mol. Biol..

[42] Pezzulo A.A., Starner T.D., Scheetz T.E., Traver G.L., Tilley A.E., Harvey B.G., Crystal R.G., McCray P.B., Zabner J. (2011). The air-liquid interface and use of primary cell cultures are important to recapitulate the transcriptional profile of in vivo airway epithelia. Am. J. Physiol. Lung Cell Mol. Physiol..

[43] Prytherch Z., Job C., Marshall H., Oreffo V., Foster M., BeruBe K. (2011). Tissue-Specific stem cell differentiation in an in vitro airway model. Macromol. Biosci..

[44] Gruenert D.C., Finkbeiner W.E., Widdicombe J.H. (1995). Culture and transformation of human airway epithelial cells. Am. J. Physiol..

[45] Leung C., Wadsworth S.J., Yang S.J., Dorscheid D.R. (2020). Structural and functional variations in human bronchial epithelial cells cultured in air-liquid interface using different growth media. Am. J. Physiol. Lung Cell Mol. Physiol..

[46] Gras D., Petit A., Charriot J., Knabe L., Alagha K., Gamez A.S., Garulli C., Bourdin A., Chanez P., Molinari N. (2017). Epithelial ciliated beating cells essential for ex vivo ALI culture growth. BMC Pulm. Med..

[47] Gras D., Bourdin A., Vachier I., de Senneville L., Bonnans C., Chanez P. (2012). An ex vivo model of severe asthma using reconstituted human bronchial epithelium. J. Allergy Clin. Immunol..

[48] Whitcutt M.J., Adler K.B., Wu R. (1988). A biphasic chamber system for maintaining polarity of differentiation of cultured respiratory tract epithelial cells. Vitro Cell Dev. Biol..

[49] Ravi M., Paramesh V., Kaviya S.R., Anuradha E., Solomon F.D. (2015). 3D cell culture systems: Advantages and applications. J. Cell Physiol.

[50] Liu G., David B.T., Trawczynski M., Fessler R.G. (2020). Advances in Pluripotent Stem Cells: History, Mechanisms, Technologies, and Applications. Stem. Cell Rev. Rep..

[51] Bolker J.A. (2017). Animal Models in Translational Research: Rosetta Stone or Stumbling Block?. Bioessays.

[52] Robinson N.B., Krieger K., Khan F.M., Huffman W., Chang M., Naik A., Yongle R., Hameed I., Krieger K., Girardi L.N. (2019). The current state of animal models in research: A review. Int. J. Surg..

[53] Kips J.C., Anderson G.P., Fredberg J.J., Herz U., Inman M.D., Jordana M., Kemeny D.M., Lotvall J., Pauwels R.A., Plopper C.G. (2003). Murine models of asthma. Eur. Respir. J..

[54] Zscheppang K., Berg J., Hedtrich S., Verheyen L., Wagner D.E., Suttorp N., Hippenstiel S., Hocke A.C. (2018). Human Pulmonary 3D Models For Translational Research. Biotechnol. J..

[55] Berube K., Prytherch Z., Job C., Hughes T. (2010). Human primary bronchial lung cell constructs: The new respiratory models. Toxicology.

[56] Ramanan V., Scull M.A., Sheahan T.P., Rice C.M., Bhatia S.N. (2014). New Methods in Tissue Engineering: Improved Models for Viral Infection. Annu Rev. Virol.

[57] Lin R.Z., Chang H.Y. (2008). Recent advances in three-dimensional multicellular spheroid culture for biomedical research. Biotechnol. J..

[58] Moroni L., de Wijn J.R., van Blitterswijk C.A. (2008). Integrating novel technologies to fabricate smart scaffolds. J. Biomater. Sci. Polym. Ed..

[59] Slaughter B.V., Khurshid S.S., Fisher O.Z., Khademhosseini A., Peppas N.A. (2009). Hydrogels in regenerative medicine. Adv. Mater..

[60] Bridges M.A., Walker D.C., Harris R.A., Wilson B.R., Davidson A.G. (1991). Cultured human nasal epithelial multicellular spheroids: Polar cyst-like model tissues. Biochem. Cell Biol..

[61] Carterson A.J., Honer zu Bentrup K., Ott C.M., Clarke M.S., Pierson D.L., Vanderburg C.R., Buchanan K.L., Nickerson C.A., Schurr M.J. (2005). A549 lung epithelial cells grown as three-dimensional aggregates: Alternative tissue culture model for Pseudomonas aeruginosa pathogenesis. Infect. Immun..

[62] Deslee G., Dury S., Perotin J.M., Al Alam D., Vitry F., Boxio R., Gangloff S.C., Guenounou M., Lebargy F., Belaaouaj A. (2007). Bronchial epithelial spheroids: An alternative culture model to investigate epithelium inflammation-mediated COPD. Respir. Res..

[63] Unsworth B.R., Lelkes P.I. (1998). Growing tissues in microgravity. Nat. Med..

[64] Castillon N., Avril-Delplanque A., Coraux C., Delenda C., Peault B., Danos O., Puchelle E. (2004). Regeneration of a well-differentiated human airway surface epithelium by spheroid and lentivirus vector-transduced airway cells. J. Gene Med..

[65] Castillon N., Hinnrasky J., Zahm J.M., Kaplan H., Bonnet N., Corlieu P., Klossek J.M., Taouil K., Avril-Delplanque A., Peault B. (2002). Polarized expression of cystic fibrosis transmembrane conductance regulator and associated epithelial proteins during the regeneration of human airway surface epithelium in three-dimensional culture. Lab. Investig..

[66] Schogler A., Blank F., Brugger M., Beyeler S., Tschanz S.A., Regamey N., Casaulta C., Geiser T., Alves M.P. (2017). Characterization of pediatric cystic fibrosis airway epithelial cell cultures at the air-liquid interface obtained by non-invasive nasal cytology brush sampling. Respir. Res..

[67] Parker J.C., Thavagnanam S., Skibinski G., Lyons J., Bell J., Heaney L.G., Shields M.D. (2013). Chronic IL9 and IL-13 exposure leads to an altered differentiation of ciliated cells in a well-differentiated paediatric bronchial epithelial cell model. PLoS ONE.

[68] Brewington J.J., Filbrandt E.T., LaRosa F.J., Moncivaiz J.D., Ostmann A.J., Strecker L.M., Clancy J.P. (2018). Generation of Human Nasal Epithelial Cell Spheroids for Individualized Cystic Fibrosis Transmembrane Conductance Regulator Study. J. Vis. Exp..

[69] Gamarra F., Bergner A., Stauss E., Stocker I., Grundler S., Huber R.M. (2006). Rotation frequency of human bronchial and nasal epithelial spheroids as an indicator of mucociliary function. Respiration.

[70] Ebsen M., Mogilevski G., Anhenn O., Maiworm V., Theegarten D., Schwarze J., Morgenroth K. (2002). Infection of murine precision cut lung slices (PCLS) with respiratory syncytial virus (RSV) and chlamydophila pneumoniae using the Krumdieck technique. Pathol. Res. Pract..

[71] Liu G., Betts C., Cunoosamy D.M., Aberg P.M., Hornberg J.J., Sivars K.B., Cohen T.S. (2019). Use of precision cut lung slices as a translational model for the study of lung biology. Respir. Res..

[72] Faisy C., Grassin-Delyle S., Blouquit-Laye S., Brollo M., Naline E., Chapelier A., Devillier P. (2014). Wnt/beta-catenin signaling modulates human airway sensitization induced by beta2-adrenoceptor stimulation. PLoS ONE.

[73] Morin C., Sirois M., Echave V., Gomes M.M., Rousseau E. (2008). EET displays anti-inflammatory effects in TNF-alpha stimulated human bronchi: Putative role of CPI-17. Am. J. Respir. Cell Mol. Biol..

[74] Carletti E., Motta A., Migliaresi C. (2011). Scaffolds for tissue engineering and 3D cell culture. Methods Mol Biol.

[75] Nichols J.E., Niles J., Riddle M., Vargas G., Schilagard T., Ma L., Edward K., La Francesca S., Sakamoto J., Vega S. (2013). Production and assessment of decellularized pig and human lung scaffolds. Tissue Eng Part. A.

[76] Petersen T.H., Calle E.A., Colehour M.B., Niklason L.E. (2012). Matrix composition and mechanics of decellularized lung scaffolds. Cells Tissues Organs.

[77] Liu Z., Anderson J.D., Deng L., Mackay S., Bailey J., Kersh L., Rowe S.M., Guimbellot J.S. (2020). Human Nasal Epithelial Organoids for Therapeutic Development in Cystic Fibrosis. Genes.

[78] Barkauskas C.E., Chung M.I., Fioret B., Gao X., Katsura H., Hogan B.L. (2017). Lung organoids: Current uses and future promise. Development.

[79] Bartfeld S., Clevers H. (2017). Stem cell-derived organoids and their application for medical research and patient treatment. J. Mol Med. (Berl).

[80] Clevers H. (2016). Modeling Development and Disease with Organoids. Cell.

[81] Sachs N., Papaspyropoulos A., Zomer-van Ommen D.D., Heo I., Bottinger L., Klay D., Weeber F., Huelsz-Prince G., Iakobachvili N., Amatngalim G.D. (2019). Long-term expanding human airway organoids for disease modeling. EMBO J..

[82] Paschini M., Kim C.F. (2019). An airway organoid is forever. EMBO J..

[83] Guimbellot J.S., Leach J.M., Chaudhry I.G., Quinney N.L., Boyles S.E., Chua M., Aban I., Jaspers I., Gentzsch M. (2017). Nasospheroids permit measurements of CFTR-dependent fluid transport. JCI Insight.

[84] Keglowich L., Roth M., Philippova M., Resink T., Tjin G., Oliver B., Lardinois D., Dessus-Babus S., Gosens R., Hostettler Haack K. (2013). Bronchial smooth muscle cells of asthmatics promote angiogenesis through elevated secretion of CXC-chemokines (ENA-78, GRO-alpha, and IL-8). PLoS ONE.

[85] Randell S.H., Fulcher M.L., O’Neal W., Olsen J.C. (2011). Primary epithelial cell models for cystic fibrosis research. Methods Mol. Biol..

[86] Comer D.M., Elborn J.S., Ennis M. (2012). Comparison of nasal and bronchial epithelial cells obtained from patients with COPD. PLoS ONE.

[87] McLellan K., Shields M., Power U., Turner S. (2015). Primary airway epithelial cell culture and asthma in children-lessons learnt and yet to come. Pediatr. Pulmonol..

[88] Batra H., Yarmus L. (2018). Indications and complications of rigid bronchoscopy. Expert Rev. Respir. Med..

[89] Soyer T. (2016). The role bronchoscopy in the diagnosis of airway disease in children. J. Thorac. Dis..

[90] Webster I., Goussard P., Gie R., Janson J., Rossouw G. (2017). The indications and role of paediatric bronchoscopy in a developing country, with high prevalence of pulmonary tuberculosis and HIV. Expert Rev. Respir. Med..

[91] Lane C., Burgess S., Kicic A., Knight D., Stick S. (2005). The use of non-bronchoscopic brushings to study the paediatric airway. Respir. Res..

[92] Doherty G.M., Christie S.N., Skibinski G., Puddicombe S.M., Warke T.J., de Courcey F., Cross A.L., Lyons J.D., Ennis M., Shields M.D. (2003). Non-bronchoscopic sampling and culture of bronchial epithelial cells in children. Clin. Exp. Allergy.

[93] Muller L., Brighton L.E., Carson J.L., Fischer W.A., Jaspers I. (2013). Culturing of human nasal epithelial cells at the air liquid interface. J. Vis. Exp..

[94] Devalia J.L., Sapsford R.J., Wells C.W., Richman P., Davies R.J. (1990). Culture and comparison of human bronchial and nasal epithelial cells in vitro. Respir. Med..

[95] Maestre-Batlle D., Pena O.M., Hirota J.A., Gunawan E., Rider C.F., Sutherland D., Alexis N.E., Carlsten C. (2017). Novel flow cytometry approach to identify bronchial epithelial cells from healthy human airways. Sci. Rep..

[96] Aydin M., Naumova E.A., Lutz S., Meyer-Bahlburg A., Arnold W.H., Kreppel F., Ehrhardt A., Postberg J., Wirth S. (2020). Do Current Asthma-Preventive Measures Appropriately Face the World Health Organization’s Concerns: A Study Presentation of a New Clinical, Prospective, Multicentric Pediatric Asthma Exacerbation Cohort in Germany. Front. Pediatr..

[97] Zhang W., Fu J., Liu J., Wang H., Schiwon M., Janz S., Schaffarczyk L., von der Goltz L., Ehrke-Schulz E., Dorner J. (2017). An Engineered Virus Library as a Resource for the Spectrum-wide Exploration of Virus and Vector Diversity. Cell Rep..

[98] Yin X., Mead B.E., Safaee H., Langer R., Karp J.M., Levy O. (2016). Engineering Stem Cell Organoids. Cell Stem. Cell.

[99] Rayner R.E., Makena P., Prasad G.L., Cormet-Boyaka E. (2019). Optimization of Normal Human Bronchial Epithelial (NHBE) Cell 3D Cultures for in vitro Lung Model Studies. Sci. Rep..

[100] Brodlie M., McKean M.C., Johnson G.E., Perry J.D., Nicholson A., Verdon B., Gray M.A., Dark J.H., Pearson J.P., Fisher A.J. (2010). Primary bronchial epithelial cell culture from explanted cystic fibrosis lungs. Exp. Lung Res..

[101] Fulcher M.L., Gabriel S., Burns K.A., Yankaskas J.R., Randell S.H. (2005). Well-differentiated human airway epithelial cell cultures. Methods Mol. Med..

[102] Iverson E., Kaler L., Agostino E.L., Song D., Duncan G.A., Scull M.A. (2020). Leveraging 3D Model Systems to Understand Viral Interactions with the Respiratory Mucosa. Viruses.

[103] Kimura H., Francisco D., Conway M., Martinez F.D., Vercelli D., Polverino F., Billheimer D., Kraft M. (2020). Type 2 inflammation modulates ACE2 and TMPRSS2 in airway epithelial cells. J. Allergy Clin. Immunol..

[104] Feng W., Guo J., Huang H., Xia B., Liu H., Li J., Lin S., Li T., Liu J., Li H. (2015). Human normal bronchial epithelial cells: A novel in vitro cell model for toxicity evaluation. PLoS ONE.

[105] Jang Y.J., Lee Y.H., Shin S.H. (2010). Rhinovirus-infected nasal polyp epithelial cells: Effect on the activation and migration of eosinophils by airborne fungi. Ann. Allergy Asthma Immunol..

[106] Broadbent L., Manzoor S., Zarcone M.C., Barabas J., Shields M.D., Saglani S., Lloyd C.M., Bush A., Custovic A., Ghazal P. (2020). Comparative primary paediatric nasal epithelial cell culture differentiation and RSV-induced cytopathogenesis following culture in two commercial media. PLoS ONE.

[107] Luengen A.E., Kniebs C., Buhl E.M., Cornelissen C.G., Schmitz-Rode T., Jockenhoevel S., Thiebes A.L. (2020). Choosing the Right Differentiation Medium to Develop Mucociliary Phenotype of Primary Nasal Epithelial Cells In Vitro. Sci. Rep..

[108] Roberts N., Al Mubarak R., Francisco D., Kraft M., Chu H.W. (2018). Comparison of paired human nasal and bronchial airway epithelial cell responses to rhinovirus infection and IL-13 treatment. Clin. Transl. Med..

[109] Jones N. (2001). The nose and paranasal sinuses physiology and anatomy. Adv. Drug Deliv. Rev..

[110] Leopold D.A. (1992). Pollution: The nose and sinuses. Otolaryngol Head Neck Surg.

[111] Jiao J., Wang C., Zhang L. (2019). Epithelial physical barrier defects in chronic rhinosinusitis. Expert. Rev. Clin. Immunol..

[112] Baturcam E., Snape N., Yeo T.H., Schagen J., Thomas E., Logan J., Galbraith S., Collinson N., Phipps S., Fantino E. (2017). Human Metapneumovirus Impairs Apoptosis of Nasal Epithelial Cells in Asthma via HSP70. J. Innate Immun..

[113] Thavagnanam S., Parker J.C., McBrien M.E., Skibinski G., Shields M.D., Heaney L.G. (2014). Nasal epithelial cells can act as a physiological surrogate for paediatric asthma studies. PLoS ONE.

[114] Vanders R.L., Hsu A., Gibson P.G., Murphy V.E., Wark P.A.B. (2019). Nasal epithelial cells to assess in vitro immune responses to respiratory virus infection in pregnant women with asthma. Respir. Res..

[115] Driehuis E., Kretzschmar K., Clevers H. (2020). Establishment of patient-derived cancer organoids for drug-screening applications. Nat. Protoc..

[116] VanDussen K.L., Marinshaw J.M., Shaikh N., Miyoshi H., Moon C., Tarr P.I., Ciorba M.A., Stappenbeck T.S. (2015). Development of an enhanced human gastrointestinal epithelial culture system to facilitate patient-based assays. Gut.

[117] Jackson D.J., Makrinioti H., Rana B.M., Shamji B.W., Trujillo-Torralbo M.B., Footitt J., Jerico D.-R., Telcian A.G., Nikonova A., Zhu J. (2014). IL-33-dependent type 2 inflammation during rhinovirus-induced asthma exacerbations in vivo. Am. J. Respir Crit. Care Med..

[118] Pech M., Weckmann M., Konig I.R., Franke A., Heinsen F.A., Oliver B., Ricklefs I., Fuchs O., Rabe K., Hansen G. (2018). Rhinovirus infections change DNA methylation and mRNA expression in children with asthma. PLoS ONE.

[119] Yamaya M., Nomura K., Arakawa K., Sugawara M., Deng X., Lusamba Kalonji N., Nishimura H., Yamada M., Nagatomi R., Kawase T. (2020). Clarithromycin decreases rhinovirus replication and cytokine production in nasal epithelial cells from subjects with bronchial asthma: Effects on IL-6, IL-8 and IL-33. Arch. Pharm Res..

[120] Lopez-Souza N., Favoreto S., Wong H., Ward T., Yagi S., Schnurr D., Finkbeiner W.E., Dolganov G.M., Widdicombe J.H., Boushey H.A. (2009). In vitro susceptibility to rhinovirus infection is greater for bronchial than for nasal airway epithelial cells in human subjects. J. Allergy Clin. Immunol..

[121] Spann K.M., Baturcam E., Schagen J., Jones C., Straub C.P., Preston F.M., Chen L., Phipps S., Sly P.D., Fantino E. (2014). Viral and host factors determine innate immune responses in airway epithelial cells from children with wheeze and atopy. Thorax.

[122] McErlean P., Favoreto S., Costa F.F., Shen J., Quraishi J., Biyasheva A., Cooper J.J., Scholtens D.M., Vanin E.F., de Bonaldo M.F. (2014). Human rhinovirus infection causes different DNA methylation changes in nasal epithelial cells from healthy and asthmatic subjects. BMC Med. Genomics.

[123] Yamaya M., Nishimura H., Deng X., Sugawara M., Watanabe O., Nomura K., Shimotai Y., Momma H., Ichinose M., Kawase T. (2020). Inhibitory effects of glycopyrronium, formoterol, and budesonide on coronavirus HCoV-229E replication and cytokine production by primary cultures of human nasal and tracheal epithelial cells. Respir. Investig..

